# Multimodal Guidewire 3D Reconstruction Based on Magnetic Field Data

**DOI:** 10.3390/s26020545

**Published:** 2026-01-13

**Authors:** Wenbin Jiang, Qian Zheng, Dong Yang, Jiaqian Li, Wei Wei

**Affiliations:** College of Electronics and Information Engineering, Suzhou University of Science and Technology, Suzhou 215000, China; 2313041005@post.usts.edu.cn (W.J.); qian.zheng@mail.usts.edu.cn (Q.Z.); yangdong97@usts.edu.cn (D.Y.); lijiaqian@mail.usts.edu.cn (J.L.)

**Keywords:** interventional surgery navigation, single view 3D reconstruction, multimodal, feature fusion

## Abstract

Accurate 3D reconstruction of guidewires is crucial in minimally invasive surgery and interventional procedures. Traditional biplanar X-ray–based reconstruction methods can achieve reasonable accuracy but involve high radiation doses, limiting their clinical applicability; meanwhile, single-view images inherently lack reliable depth cues. To address these issues, this paper proposes a multimodal guidewire 3D reconstruction approach that integrates magnetic field information. The method first employs the MiDaS v3 network to estimate an initial depth map from a single image and then incorporates tri-axial magnetic field measurements to enrich and refine the spatial information. To effectively fuse the two modalities, we design a multi-stage strategy combining nearest-neighbor matching (KNN) with a cross-modal attention mechanism (Cross-Attention), enabling accurate alignment and fusion of image and magnetic features. The fused representation is subsequently fed into a PointNet-based regressor to generate the final 3D coordinates of the guidewire. Experimental results demonstrate that our method achieves a root-mean-square error of 2.045 mm, a mean absolute error of 1.738 mm, and a *z*-axis MAE of 0.285 mm on the test set. These findings indicate that the proposed multimodal framework improves 3D reconstruction accuracy under single-view imaging and offers enhanced visualization support for interventional procedures.

## 1. Introduction

Cardiovascular and cerebrovascular interventions have become efficient and minimally invasive treatment strategies in modern clinical practice. However, such procedures heavily rely on real-time navigation using two-dimensional (2D) X-ray images provided by digital subtraction angiography (DSA) [1,2,3]. This reliance not only limits clinicians’ ability to accurately assess the three-dimensional (3D) trajectory of guidewires and catheters, but also exposes both patients and surgeons to prolonged high-dose X-ray radiation due to continuous fluoroscopy. Long-term exposure may cause radiation-related health risks, including cellular damage, tissue lesions, and even increased cancer risk [4]. Therefore, reducing radiation dose while maintaining sufficient image quality has become a critical challenge in the development of interventional surgical technologies [5]. Achieving 3D reconstruction of guidewires can provide accurate spatial information, substantially improving procedural safety and precision [6,7]. It enables clinicians to visualize guidewire paths more intuitively, reduces the likelihood of incorrect manipulation, and offers technical support for emerging intervention paradigms such as intelligent navigation and remote robotic surgery.

Traditional guidewire reconstruction methods typically depend on biplane or multi-view C-arm X-ray imaging, where 3D shape recovery is accomplished through geometric triangulation or stereo matching [8]. However, these approaches require multiple fluoroscopic acquisitions, leading to increased radiation exposure and higher operational costs. An alternative strategy embeds a permanent magnet into the guidewire tip to track its position, but such hardware modifications are impractical for routine clinical use [9].

To address these issues, this study proposes a novel single-view guidewire 3D reconstruction method enhanced by magnetic-field sensing. This method can achieve accurate pose estimation from monocular DSA images using only magnetic guidewires, without additional radiation or the need for multi-view acquisition. The framework consists of two major components: a multimodal feature fusion module and a PointNet-based 3D coordinate regression module.

First, MiDaS v3 [10] is employed to estimate coarse depth from the image. Meanwhile, sparse tri-axial magnetic-field measurements are interpolated and encoded into continuous high-dimensional representations to compensate for depth ambiguities in complex regions where image-based estimation alone may fail.

Since visual and magnetic-field modalities exist in inherently different physical domains—with significant disparities in scale, distribution, and noise characteristics—direct fusion leads to severe feature misalignment. To overcome this, we introduce a multi-stage fusion strategy combining K-Nearest Neighbors (KNN) matching and a cross-modal attention mechanism [11]. Specifically, depth representations from both modalities are first projected into a unified high-dimensional embedding space. Then, for each magnetic-field feature, KNN search is performed within the image-feature space to identify the most similar neighboring embeddings, establishing initial correspondences and capturing local structural cues. Subsequently, a cross-modal attention module is applied, where magnetic-field embeddings serve as queries, and image embeddings act as keys and values. Through explicit attention computation, the module facilitates dynamic information exchange and structural alignment across modalities, generating a unified and semantically consistent fused feature representation.

Finally, the fused features are fed into a PointNet-based point-wise 3D coordinate regressor [12,13]. This module encodes local features via multilayer perceptrons (MLPs), aggregates global contextual information through max pooling, and concatenates local–global features to achieve accurate 3D coordinate regression, yielding the full guidewire geometry in the real-world coordinate system.

## 2. Related Work

In recent years, extensive research efforts have been devoted to the 3D reconstruction of guidewires in interventional procedures. Most existing studies rely on either biplane or single-plane imaging. Burgner et al. [14] proposed a 3D guidewire reconstruction method based on biplane image sequences, where guidewire positions are simultaneously tracked across two fluoroscopic views to achieve 3D visualization within vascular structures. Although effective, this method requires high-quality image sequences and involves significant effort in data acquisition. Hoffmann et al. [15] employed graph-based search techniques to identify catheter segments in each view during electrophysiology (EP) procedures and integrated them into a 3D model. While their approach performs well on straight catheter segments, noticeable errors remain in highly curved regions, particularly near the catheter tip. Wagner et al. [16] adopted a dual-threshold segmentation strategy combined with Gaussian fitting of image histograms to detect guidewire regions. Using epipolar geometry, point correspondences were established between stereoscopic views, and 3D centerlines were generated by computing intersections or closest-point pairs between corresponding 2D centerline points. Although dual-view reconstruction improves geometric accuracy, it places stringent requirements on image quality and increases radiation exposure during clinical practice.

To reduce radiation dose, Breininger et al. [17] introduced a single-view rigid guidewire reconstruction method that applied homomorphic and bilateral filtering to X-ray images, and reconstructed 3D shape using epipolar constraints derived from real and virtual guidewire positions. Jianu et al. [18] generated simulated single-view fluoroscopic images using the CathSim simulator and trained the 3D-FGRN network to recover 3D guidewire geometry. Their method used Huber loss and regularization terms to ensure prediction accuracy and structural consistency. With advances in deep learning, monocular depth estimation networks have been widely adopted in image analysis [8], offering new perspectives for single-plane guidewire reconstruction. These methods infer relative depth from a single fluoroscopic frame and utilize it to recover 3D structure. To compensate for intrinsic scale and translation ambiguities in monocular geometry, additional loss functions or structural constraints are typically introduced. Compared with traditional biplane methods, depth-estimation-based approaches substantially reduce imaging requirements and enhance deployment flexibility.

Beyond purely visual approaches, researchers have also explored multimodal strategies that integrate magnetic, ultrasound, or optical sensing with imaging to enable robust 3D reconstruction [19,20]. Among these modalities, magnetic sensing has attracted considerable attention due to its insensitivity to occlusion and ability to operate under non-line-of-sight conditions. Existing magnetic tracking systems typically employ tri-axial magnetic sensors to measure magnetic field intensities, and estimate sensor position and orientation based on dipole field models or magnetic attenuation characteristics [21,22]. However, magnetic measurements are susceptible to interference from metallic environments, electromagnetic noise, and inherent sensor nonlinearity, leading to degraded localization accuracy. Moreover, the mapping from magnetic field to spatial position/curvature is nonlinear and often underdetermined, requiring calibrated field models or physical constraints for stable solutions. Zhao et al. [23] proposed a hybrid reconstruction framework for flexible magnetic guidewires that integrates magnetic field modeling with image-based segmentation and skeleton extraction from single-plane fluoroscopy. Physical constraints were further incorporated to optimize the recovered guidewire morphology. Such multimodal fusion approaches leverage complementary sensing advantages and exhibit strong robustness in low-texture or occluded regions, representing an important development direction for guidewire 3D reconstruction.

While single-view methods reduce radiation exposure and simplify hardware requirements, reconstruction accuracy often deteriorates in the proximal segments of the guidewire, particularly in regions with high curvature, where prediction errors tend to be more pronounced [24].

## 3. Methods

**Figure 1:**
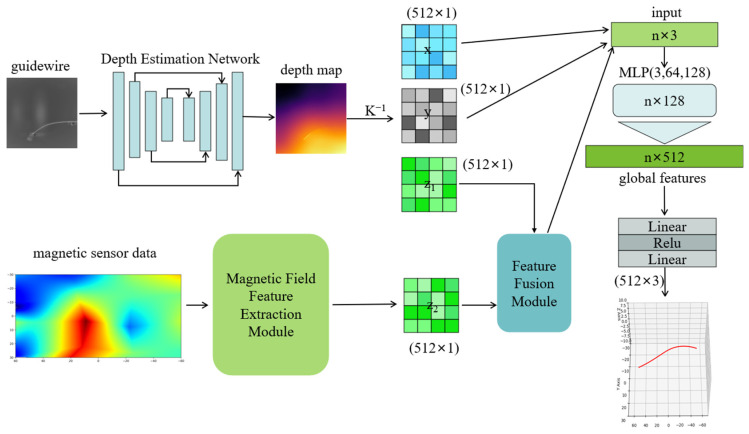
Overall Architecture of the Proposed Model.

This study proposes a single-plane guidewire 3D reconstruction method that integrates image-based depth estimation with magnetic-field sensing. The overall framework, illustrated in Figure 1, consists of four stages: image depth estimation, magnetic field feature extraction module, feature fusion module, and 3D coordinate regression.

First, the MiDaS v3 network is employed to estimate an initial depth map from a single fluoroscopic image, from which a sparse point cloud is constructed to represent the coarse 3D geometry of the guidewire. Second, tri-axial magnetic induction signals are collected using magnetic sensors placed around the guidewire. The measured magnetic field is interpolated and smoothed to enhance spatial continuity, and then encoded into a high-dimensional representation through a feature encoder.

In the feature fusion module, we adopt a hybrid strategy combining K-nearest-neighbor (KNN) retrieval with a cross-modal attention mechanism. KNN establishes local correspondences between image and magnetic-field embeddings, while cross-modal attention enables dynamic interaction and structural alignment across modalities, yielding a unified fused representation.

### 3.1. Feature Construction and Encoding

The guidewire studied in this work is a slender structure characterized by continuous shape variations and non-uniform curvature, and therefore its spatial distribution must first be accurately extracted from the image. To achieve this, we employ the MiDaS v3 network as the depth feature extractor for the image modality. MiDaS v3 takes a single guidewire image as input and produces an initial depth estimation for the guidewire region. As illustrated in Figure 1, the MiDaS v3 architecture consists of an encoder, a decoder, and a multi-scale feature fusion module, and outputs a depth map with relative scale. Because the predicted depth map provides only scale-ambiguous relative depth, an additional transformation is required to recover the guidewire’s geometry in real 3D space. To this end, we first use the known camera intrinsic matrix K, and then apply the back-projection formulation in Equation (1) to map each pixel and its corresponding depth value into a 3D point in the camera coordinate system. This yields a point set $p_{i}=(x_{i},y_{i},z_{i})\in R^{3}$, representing the reconstructed point cloud of the guidewire.

(1) $$\left[\begin{array}{l} X\\ Y\\ Z \end{array}\right]=D(x,y)\cdot K^{-1}\left[\begin{array}{l} x\\ y\\ 1 \end{array}\right]$$

**Figure 2:**
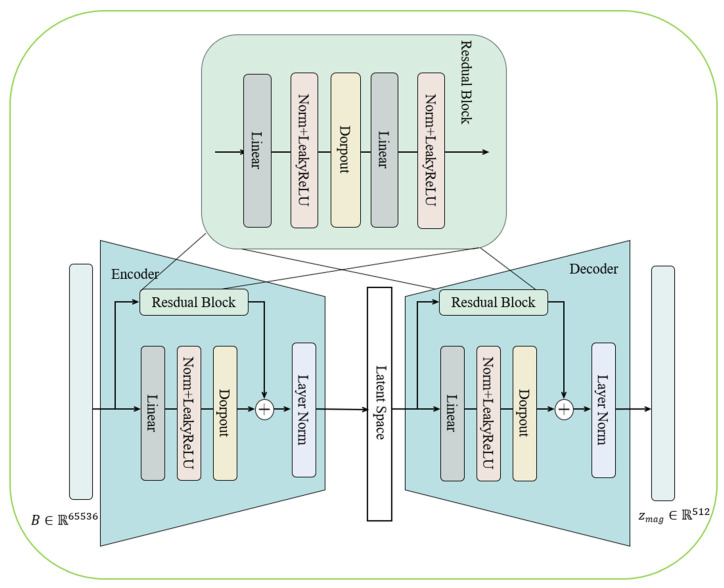
Magnetic field feature extraction module.

However, the depth estimated by MiDaS v3 does not possess a true metric scale and only reflects relative ordering along the viewing direction. To compensate for this limitation, we further introduce the magnetic-field modality to perform absolute scale correction. From each magnetic sensor, we obtain the three-axis components $B_{x}$, $B_{y}$, $B_{z}$ along the x-, y-, and z-axes, and compute the corresponding magnetic flux density B. Since the magnetic field strength is physically correlated with the distance to the magnetic source—such as in the magnetic dipole model where $B\propto 1/r^{3}$—the magnetic modality provides complementary information that is inherently consistent with real-world spatial scales. To effectively leverage this physical property, we design a neural-network-based depth correction module that maps magnetic measurements to an absolute depth component for each point in the 3D point cloud. As shown in Figure 2.

The proposed model consists of two main components: a Depth Encoder and a Depth Decoder. Let the input be the flattened magnetic-field vector $B_{total}\in R^{65536}$, and the output be the corrected depth component $z_{mag}$, The mapping from $B_{total}\in R^{65536}$ to $z_{mag}$ is defined by Equation (2):

(2) $$z_{mag}=f(B_{total})=f_{dec}(f_{enc}(B_{total}))$$

In this module, the encoder $f_{enc}$ compresses the high-dimensional magnetic-field features into a low-dimensional latent representation $z\in R^{256}$, which is then $f_{dec}$ decoded to reconstruct the 3D point cloud structure. Each layer is composed of a linear transformation followed by batch normalization (BatchNorm) and a nonlinear activation function (LeakyReLU).

(3) $$\phi_{i}(B_{total})=\sigma_{i}(BN_{i}(W_{i}B_{total}+b_{i}))$$

The entire network is formed by a series of stacked layers $\phi_{i}$, which can be expressed as:

(4) $$f(B_{total})=(\phi_{L}\circ \phi_{L-1}\circ \cdot \cdot \cdot \circ \phi_{1})(B_{total})$$

The final output $z_{mag}$ is further normalized to ensure consistent geometric scaling. The magnetic-field feature extraction network is designed to reconstruct the local spatial structure, and employs the Chamfer Distance (CD) as the loss function to measure the geometric discrepancy between the predicted point cloud and the ground-truth point cloud.

### 3.2. Data Fusion and Regression

**Figure 3:**
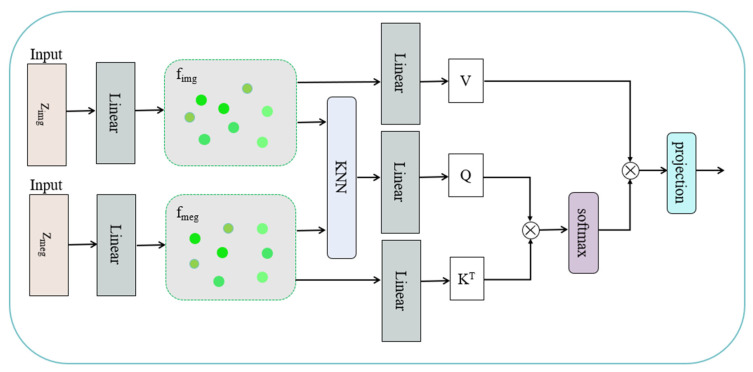
Feature fusion module.

To enable effective complementarity between the image modality and the magnetic-field modality in the depth estimation task, we propose a multi-stage fusion strategy that integrates k-nearest neighbor (KNN) matching with a cross-modal attention mechanism. Guided by the depth estimates $z_{mag}\in R^{B\times N\times 1}$ provided by the magnetic modality, this strategy aligns and fuses the embedded representations of the image-derived depth $z_{img}\in R^{B\times N\times 1}$. The overall architecture of the proposed fusion framework is illustrated in Figure 3.

The dual-branch embedding module maps the original scalar depth values into a high-dimensional feature space, thereby unifying the representations of the two modalities—image-derived depth and magnetic-field-derived depth. This module consists of two independent linear transformation layers applied separately to the image modality and the magnetic modality, producing two embeddings of equal dimensionality, denoted as $f_{img},f_{mag}\in R^{D}$. Subsequently, the two modality-specific embeddings are concatenated along the channel dimension and passed through a fusion mapping layer to further integrate the complementary information:

(5) $$f_{fuse}=W_{fuse}[f_{img};f_{mag}]+b_{fuse}$$

“[⋅;⋅]” denotes channel-wise concatenation, and $W_{fuse}\in R^{F\times 2D}$ and $b_{fuse}\in R^{F}$ are learnable parameters of the fusion layer, producing the output $f_{fuse}\in R^{F}$. To further enhance the local matching capability of the fused features, we introduce a neighborhood search mechanism based on K-Nearest Neighbors (KNN). Specifically, for each magnetic-field embedding vector $f_{mag,i}$, we compute the Euclidean distances to all image embeddings $\left\{f_{img,j}\left|j=1,2\ldots ,N\right.\right\}$ and select the k nearest neighbors:

(6) $$Z_{knn}=KNN\left(f_{mag}^{\left(i\right)},\left\{f_{img}^{\left(j\right)}\right\}\right)$$

The neighborhood information not only provides initial pairings for the subsequent attention mechanism but also facilitates local contextual enhancement of the features. After achieving unified embeddings and preliminary fusion of the two modalities, we further introduce a cross-attention mechanism to establish dynamic relationships between the magnetic-field and image features. This enhances cross-modal interaction and matching representation, enabling information completion and feature refinement. Specifically, given the fused feature representation $f_{fuse}$, the query (Q), key (K), and value (V) vectors are constructed as follows:

(7) $$Q=W_{Q}\cdot f_{mag},K=W_{K}\cdot f_{img},V=W_{V}\cdot f_{img}$$

$W_{Q},W_{K},W_{V}\in R^{F\times F}$ are learnable parameter matrices $Q,K,V\in R^{N\times F}$. Next, the attention weights of the magnetic-field features with respect to the image features are computed as:

(8) $$A=Soft\max\left(\frac{Q\cdot K^{T}}{\sqrt{F}}\right)\in R^{N\times N}$$

(9) $$Z_{attn}=A\cdot V\in R^{N\times F}$$

$Z_{attn}$ denotes the attention output after fusion, which dynamically aggregates the matched features from both image and magnetic-field modalities. To further enhance the geometric consistency and local perceptual capability of the predicted depths, we employ a PointNet-style per-point regression network. This architecture preserves point-wise independence while capturing global contextual information, enabling high-precision depth regression for each point.

Specifically, the features obtained from the cross-attention fusion $Z_{attn}$ are concatenated with the KNN-selected image neighborhood features $Z_{knn}$ to form the final input for regression. Each concatenated point feature is then processed by a shared multi-layer perceptron (MLP) to extract local representations:

(10) $$f_{a}=MLP_{local}(\left[Z_{attn};Z_{knn}\right])\in R^{128}$$

After extracting the local features, a global feature enhancement mechanism is applied. Specifically, the local features of all points $f_{a}$ are aggregated via a max-pooling operation to obtain a global feature $f_{g}$. The global feature is then concatenated back to each point as an enhanced representation, which is fed into the regression head to predict the 3D coordinates of each point:

(11) $$\hat{p_{i}}=MLP_{reg}\left(f_{a},f_{g}\right)$$

The MLP regression module consists of two linear layers with a nonlinear activation function applied in between. The output $\hat{p_{i}}=\left(\hat{x_{i}},\hat{y_{i}},\hat{z_{i}}\right)$ represents the predicted 3D coordinates of the points. The point cloud regression network is trained to accurately predict the 3D coordinates, using a weighted mean absolute error (WMAE) as the loss function.

(12) $$L{\mathrm{oss}}_{WMAE}=\frac{1}{N}\sum_{i=1}^{N}\left(w_{x}\left|x_{i}-\hat{x_{i}}\right|+w_{y}\left|y_{i}-\hat{y_{i}}\right|+w_{z}\left|z_{i}-\hat{z_{i}}\right|\right)$$

The errors along the x and y axes are assigned weights of w_x_ = 1 and w_y_ = 1, while the error along the z axis is given a higher weight of w_z_ = 5. This increased weighting along the depth direction is motivated by the following considerations: in the guidewire 3D reconstruction task, deviations along the vertical (z) axis have a more pronounced impact on the overall spatial geometry, and the discrepancies between the magnetic-field and image modalities are more sensitive in the depth dimension. By assigning a larger weight to w_z_, the optimization process emphasizes depth prediction, thereby improving the spatial consistency and geometric accuracy of the reconstructed 3D structure.

## 4. Experiments

### 4.1. Experimental Environment Setup

**Figure 4:**
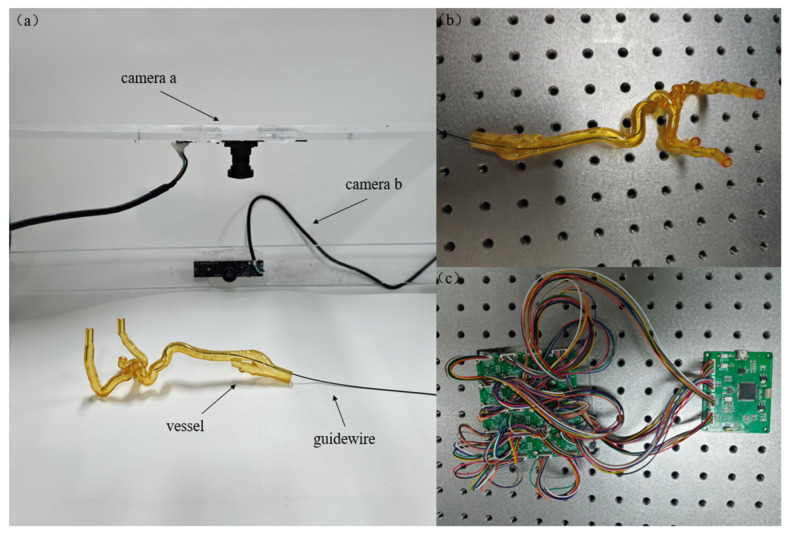
(**a**) Simulation platform. (**b**) Vascular phantom. (**c**) Tri-axial magnetic sensor array.

This study used a self-built guidewire data acquisition platform for data collection, as shown in Figure 4a. The platform mainly consists of a bionic system, a high-precision binocular camera setup, and a magnetic sensor array (AK09940A) at the base. The bionic system shown in Figure 4b uses transparent silicone vascular channels, replicating the structure and pathways of human arteries, providing a controllable and visible environment for in vitro guidewire operations (it should be noted that during actual data collection, the vascular model was removed, because the refraction of the transparent vessel walls would alter the direction of light, causing local deformation or uneven brightness at the edges of the guidewire captured by the camera; thus, in the experiment, the guidewire was displayed in common shapes directly). The binocular camera system synchronously captures image sequences of the guidewire moving inside the vessel, providing high-resolution visual information. Meanwhile, the magnetic sensor array installed at the base records the magnetic field modality data of the guidewire, supplementing depth and directional cues that are difficult to obtain from visual information alone. The layout of the magnetic sensor array is shown in Figure 4c.

During dataset construction, multiple simulated vitro guidewire manipulations were performed using a binocular orthogonal setup. Specifically, two high-definition cameras were positioned perpendicularly to form an orthogonal stereo system for capturing the guidewire’s trajectory. It is worth noting that during certain acquisition stages, the transparent silicone vascular model was temporarily removed to prevent occlusion of the guidewire. Even in the absence of the vascular model, the spatial shape of the guidewire was controlled by the operator through its insertion direction, applied force, and bending tendency, thus still forming continuous 3D structures with varying curvature segments. This approach ensured sufficient diversity in the training data and the feasibility of algorithm validation.

To establish the mapping between image-plane coordinates and spatial coordinates, the binocular camera system was geometrically calibrated using a standard checkerboard, yielding intrinsic parameters, distortion coefficients, and the extrinsic relationship between the two cameras. Ultimately, 282 pairs of vertically oriented stereo images with a resolution of 2048 × 1536 were captured, along with their corresponding 3D labeled coordinates. Concurrently, the 4 × 4 magnetic sensor array at the base of the platform recorded the guidewire’s magnetic-field features for each image frame. The magnetic data mainly consist of three-axis components (B_x_, B_y_, and B_z_) that encode the relative vertical distance between the guidewire and the magnetic source, providing additional information for depth (*z*-axis) estimation. The measured signals originate from magnetic sources fixed beneath the platform, which produce a spatially stable magnetic field suitable for constructing reliable multimodal depth constraints. The corresponding magnetic-field measurements were integrated as an additional modality alongside the image data to form the final dataset.

Although the ultimate application scenario of this study involves X-ray fluoroscopic images, from the perspective of 3D reconstruction algorithms, the core task is to recover 3D information from 2D projections. Both visible-light cameras and X-ray imaging adhere to similar perspective projection geometry. Therefore, the primary focus of this study is to validate the effectiveness of the core idea: “leveraging magnetic-field information to compensate for depth ambiguity in single-view imaging.”

### 4.2. Experimental Setup

Prior to model training, the raw images were preprocessed. The original images have a resolution of 2048 × 1536, which is non-square and not suitable for uniform model input. To address this, a two-step preprocessing strategy was adopted. First, the images were symmetrically padded along the height dimension (256 pixels at both the top and bottom), resulting in a square image of 2048 × 2048 while preserving the original width. Next, the padded images were resized to 512 × 512 using bilinear interpolation to standardize the resolution for subsequent model training.

**Figure 5:**
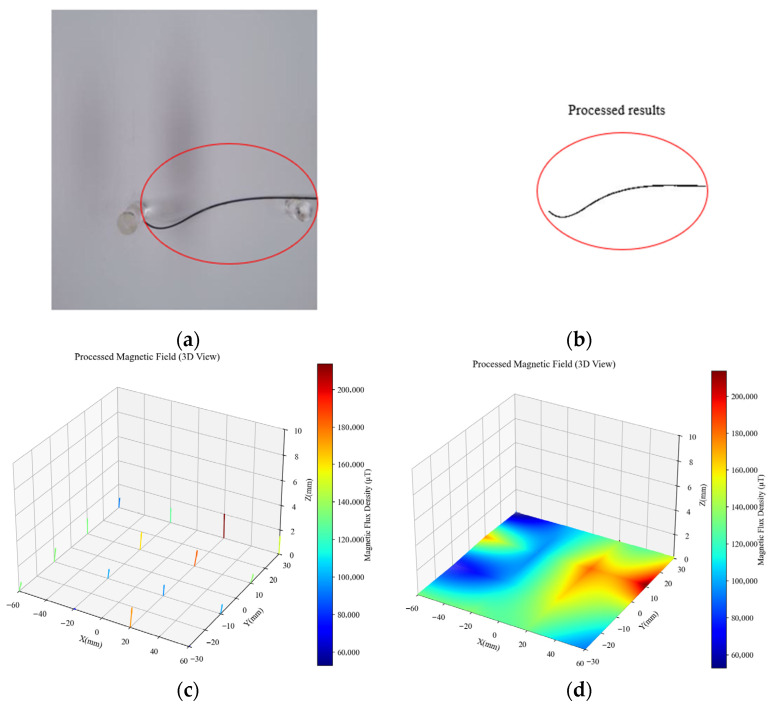
(**a**) Original image. (**b**) Thresholded image. (**c**) Original magnetic field. (**d**) Processed magnetic field.

To extract the guidewire region, a coarse segmentation based on intensity thresholding was applied, exploiting the low grayscale and slender shape of the guidewire in the image. This step effectively suppresses background interference and improves the accuracy of subsequent depth estimation, as illustrated in Figure 5b. Figure 5c shows the magnetic-field data captured by the 4 × 4 sensor array, where the arrow directions indicate the local magnetic-field orientation, and the color intensity represents the magnetic flux magnitude (red for stronger intensity, blue for weaker).

To address the sparsity and uneven distribution of the magnetic-field data, a 3D interpolation using the RegularGridInterpolator from the scipy library was employed. This method constructs an interpolator based on a regular grid and supports both linear and nearest-neighbor interpolation, enabling continuous estimation of magnetic-field values between measured points. The interpolated magnetic-field data are shown in Figure 5d, representing the feature distribution within the region.

For data augmentation, the original 282 samples were expanded to 1128 samples using horizontal flipping, rotation, and scaling. The dataset was then split into training and validation sets at a 7:3 ratio for initial model training, where the network maps the processed magnetic-field information to the corresponding guidewire depth. The model was trained with a batch size of 4 for 200 epochs using the Adam optimizer, with a learning rate of 1 × 10^−4^ and a weight decay of 1 × 10^−4^.

Subsequently, a multi-stage fusion strategy integrating k-nearest neighbor (KNN) matching and cross-modal attention (Cross-Attention) was applied to efficiently align and fuse the magnetic-field features—particularly the depth component (*z*-axis)—with the image-derived depth features. The fused features were then input into the regression model for training. For this stage, the dataset was split into training, validation, and test sets at a 7:2:1 ratio, with a batch size of 4 and 200 training epochs, using the Adam optimizer with a learning rate of 1 × 10^−3^. The final trained model was evaluated on an independent test set (10% of the data) to assess its generalization performance on unseen samples.

## 5. Experimental Results and Analysis

### 5.1. Evaluation Criteria and Metrics

To comprehensively assess the performance of the model, three commonly used error metrics were employed. Root Mean Square Error (RMSE) quantifies the square root of the mean squared differences between predicted and ground-truth values. RMSE is particularly sensitive to large errors and effectively reflects their impact on overall performance. Mean Absolute Error (MAE) calculates the average absolute difference between predictions and ground-truth values, providing a measure of the overall prediction accuracy. Hausdorff Distance (HD) measures the maximum deviation between two point sets, emphasizing the spatial consistency and shape alignment between the predicted and ground-truth point clouds.

(13) $$RMSE=\sqrt{\frac{1}{N}{\sum_{i=1}^{N}\left(d_{i}-\hat{d_{i}}\right)}^{2}}$$

(14) $$MAE=\frac{1}{N}\sum_{i=1}^{N}{\left\|P_{i}-\hat{P_{i}}\right\|}_{2}=\frac{1}{N}\sum_{i=1}^{N}\sqrt{{\left(x_{i}-\hat{x_{i}}\right)}^{2}+{\left(y_{i}-\hat{y_{i}}\right)}^{2}+{\left(z_{i}-\hat{z_{i}}\right)}^{2}}$$

$y_{i}$ and $\hat{y_{i}}$ denote the ground-truth and predicted values, respectively, and N is the total number of samples. Specifically, $P_{i}=\left(x_{i},y_{i},z_{i}\right)$ represents the true 3D point coordinates, while $\hat{P_{i}}$ corresponds to the predicted points.

For two point sets $A=\left\{a_{1},a_{2},a_{3},\ldots ,a_{n}\right\}$ and $B=\left\{b_{1},b_{2},b_{3},\ldots ,b_{n}\right\}$, the bidirectional Hausdorff distance is defined as follows:

(15) $$HD\left(A,B\right)=\max\left\{\underset{a\in A}{\sup}\underset{b\in B}{\inf}\left\|a-b\right\|,\underset{b\in B}{\sup}\underset{a\in A}{\inf}\left\|b-a\right\|\right\}$$

where $\left\|\cdot \right\|$ denotes the Euclidean distance between points.

### 5.2. Results and Analysis

**Figure 6:**
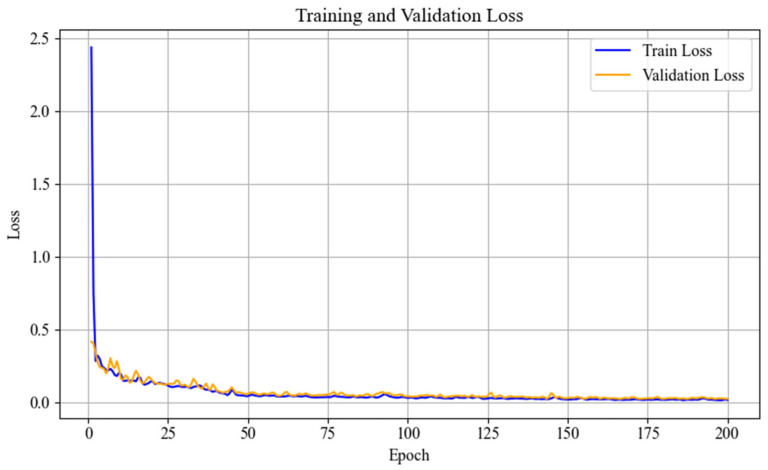
Model training and validation loss.

The loss curves for the guidewire point cloud regression network during training are shown in Figure 6. From the training and validation loss curves, it can be observed that the model converges rapidly during the initial stage (the first five epochs), with the training error dropping from 2.3 to approximately 0.2 and the validation error decreasing from 0.37 to 0.16. This demonstrates that the model achieves a good initial fit and exhibits a reasonable degree of adaptability to the validation set. During the subsequent training process, the validation loss fluctuates slightly but remains stable, with the overall trend approaching convergence. No obvious signs of overfitting are observed, indicating that the model maintains good stability throughout training.

**Figure 7:**
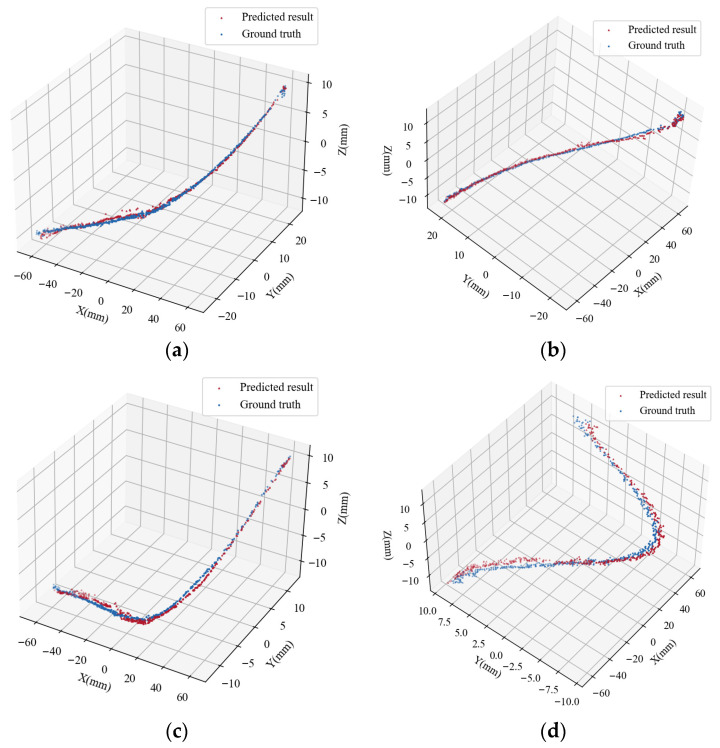
Predicted and real comparison results of four different forms of guidewires. (**a**) left-bending guidewire; (**b**) right-bending guidewire; (**c**) guidewire with larger left curvature; (**d**) S-shaped guidewire.

To validate the effectiveness of the proposed depth-fusion model for guidewire 3D point cloud reconstruction, several representative samples from the test set were selected, and their predicted point clouds were visualized and overlaid with the corresponding ground-truth point clouds. In the figures, red triangles indicate the model predictions, while blue circles represent the ground-truth points. Figure 7 presents comparisons of predicted and ground-truth 3D point clouds for four typical samples.

**Figure 8:**
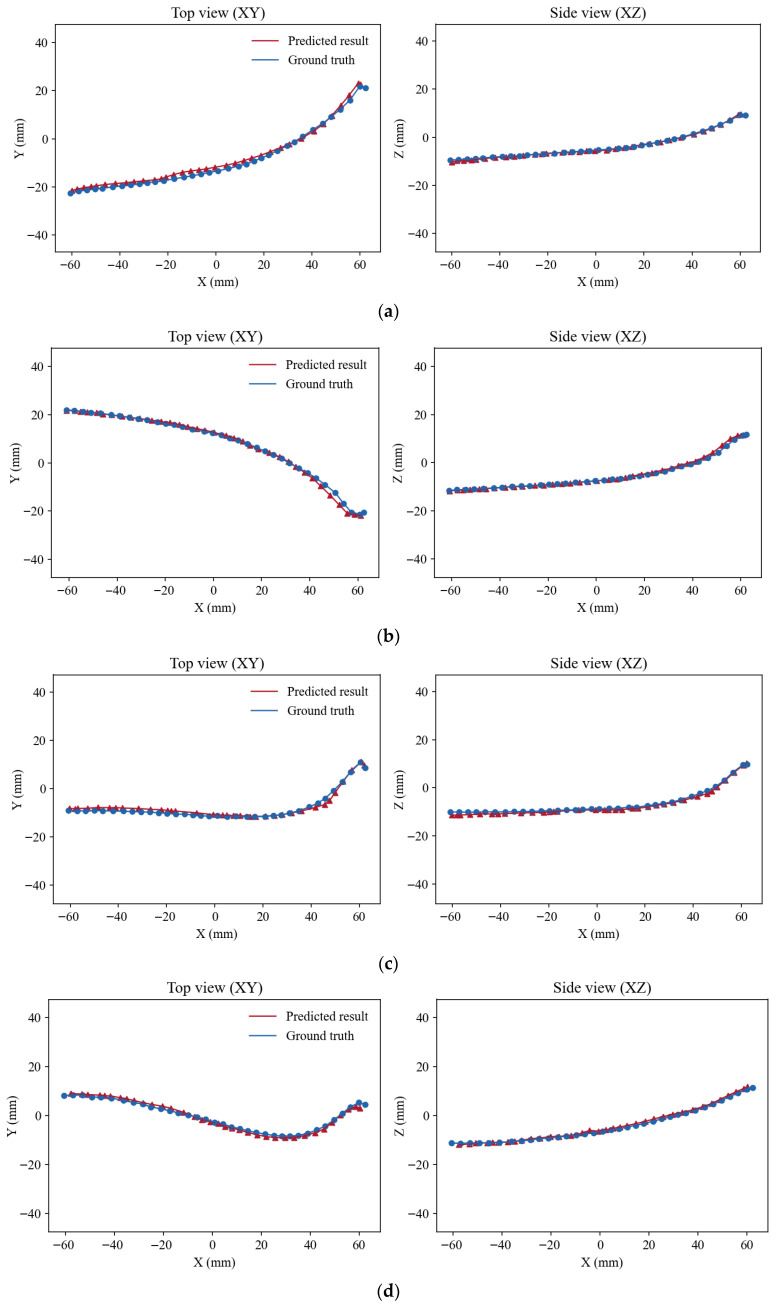
Top (XY) and side (XZ) views comparing predicted and ground-truth guidewires of four different shapes. (**a**) left-bending guidewire; (**b**) right-bending guidewire; (**c**) guidewire with larger left curvature; (**d**) S-shaped guidewire.

To further facilitate intuitive geometric comparison, Figure 8 illustrates the corresponding top (XY) and side (XZ) views of the predicted and ground-truth guidewires. From these planar projections, it can be observed that the predicted guidewire centerlines closely follow the ground-truth trajectories in both lateral and depth directions. In particular, the alignment remains consistent along the guidewire’s main body as well as in curved or bending regions, indicating that the proposed model effectively preserves both global shape and local geometric variations.

Visual inspection reveals that, for basic shapes, the predicted points align closely with the ground-truth point clouds, particularly in key regions such as the guidewire’s main body or bending segments, demonstrating high geometric consistency. Notably, due to the incorporation of magnetic-field modality depth information, the predicted point clouds exhibit good local continuity and overall contour fidelity. However, a small number of outliers can still be observed, manifesting as isolated points distant from the main structure. These abnormal predictions primarily occur at image edges or in regions with weak texture.

Quantitative analysis of the 3D reconstruction errors was also conducted from multiple perspectives. Table 1 compares the performance of several mainstream monocular depth estimation methods on the guidewire 3D reconstruction task, including Adabins [25], SharpNet [26], MiDaS [27], ZoeDepth [28], MiDaS v3 [10], and the proposed method. Incorporating magnetic-field information significantly improves reconstruction accuracy. Compared with the baseline model using only MiDaS v3 image depth, the fused model reduces the root mean square error (RMSE) from 2.842 mm to 2.045 mm, a decrease of approximately 28.0%; the mean absolute error (MAE) decreases from 2.234 mm to 1.738 mm, a reduction of about 22.2%.

Especially in the depth direction, the mean absolute error along the *z*-axis (MAE_z) decreases dramatically from 1.325 mm to 0.285 mm, a reduction of 78.5%, indicating that the incorporation of magnetic-field information significantly enhances the model’s depth perception. Furthermore, the Hausdorff distance (HD), which reflects the worst-case boundary error, is reduced from 8.754 mm to 7.603 mm (approximately 13.1% improvement), further demonstrating that the fused model is more robust in reconstructing spatial boundaries.

It is also worth noting that MiDaS v3 outperforms ZoeDepth [28] in 3D reconstruction accuracy, particularly along the depth axis (MAE_z), with an improvement of approximately 37.3%. After further integrating magnetic-field information, the proposed model achieves significant gains across all metrics, fully validating the importance of the magnetic-field modality in enhancing guidewire 3D reconstruction accuracy.

### 5.3. Ablation Studies

This study uses a traditional encoder–decoder architecture as the baseline and designs a multimodal fusion regression model that integrates magnetic-field and image depth features. To further improve guidewire depth estimation and 3D reconstruction accuracy, a k-nearest neighbor (KNN) feature fusion module and a cross-modal attention (Cross-Attention) module were incorporated. To validate the effectiveness of each module, ablation experiments were conducted on the self-constructed guidewire dataset, as summarized in Table 2. The results show that the performance of the model improves progressively as different modules are added. The baseline encoder–decoder model, when used alone for feature fusion and regression, achieves limited performance in terms of RMSE, MAE, and MAE_z. With the addition of the KNN feature fusion module, the model can effectively match the depth responses of the magnetic-field and image features within local neighborhoods. This results in a significant reduction in the depth-direction error (MAE_z), indicating that the module enhances local spatial correlations. When the cross-modal attention mechanism is further incorporated, the model can adaptively capture feature dependencies between the two modalities at a global level. Consequently, the overall RMSE and MAE metrics decrease, demonstrating the effectiveness of integrating these two modules into the network.

## 6. Conclusions

In this work, we present a single-view guidewire 3D reconstruction framework that integrates image-based depth estimation with magnetic-field modality information. To mitigate the inherent limitations of traditional monocular approaches, which suffer from insufficient depth observability, the proposed method fuses image depth predictions obtained from the MiDaS v3 network with local physical signals acquired from magnetic-field sensors. Through feature alignment and cross-modal attention mechanisms, the model is able to exploit complementary information from both modalities, enabling accurate regression of guidewire point clouds under single-view conditions.

Experimental results obtained in a controlled laboratory environment with optical imaging demonstrate that the proposed fusion regression model achieves improved accuracy and robustness across multiple evaluation metrics. In particular, notable performance gains are observed in depth estimation along the *z*-axis, as reflected by reduced MAE, RMSE, and Hausdorff Distance compared with image-only baselines. These results indicate that incorporating magnetic-field information effectively compensates for depth ambiguity and spatial sparsity that commonly arise in single-view reconstruction scenarios.

It should be noted that the current validation is limited to laboratory experiments and does not directly reflect clinical imaging conditions. Future work will therefore focus on several specific directions, including extending the method to larger-scale and noisier magnetic-field datasets, adapting the architecture to handle guidewires with more complex geometries, and evaluating performance on imaging data that more closely resemble clinical X-ray or DSA environments. Such studies are necessary to further assess the potential of the proposed multimodal fusion approach for use in practical interventional navigation and robotic-assisted procedures.

## Figures and Tables

**Table 1:** Experimental comparison results.

	RMSE (mm)	MAE (mm)	MAE_z (mm)	HD (mm)
Adabins [25]	4.193	3.528	3.17	13.612
SharpNet [26]	3.982	3.074	2.315	11.912
MiDaS [27]	3.757	3.157	2.721	12.802
ZoeDepth [28]	3.112	2.784	2.113	10.545
MiDaS v3 [10]	2.842	2.234	1.325	8.754
MiDaS v3 + magnetic field	**2.045**	**1.738**	**0.285**	**7.603**

**Table 2:** Ablation study with different modules introduced.

Base	KNN	Cross-Attention	RMSE (mm)	MAE (mm)	MAE_z (mm)
√	×	×	3.184	2.816	0.563
√	√	×	2.554	2.223	0.292
√	×	√	2.128	1.828	0.407
√	√	√	**2.045**	**1.738**	**0.285**

√ and × indicate whether the corresponding module is used or not, respectively.

## Data Availability

The original contributions presented in this study are included in the article. Further inquiries can be directed to the corresponding author.

## References

[1] Li N., He J.A., Chen Y., Zhou S. (2022). A Survey on the Progress of Computer-Assisted Vascular Intervention. J. Comput. Aided Des. Graph..

[2] Zhao Y., Mei Z., Luo X., Mao J., Zhao Q., Liu G., Wu D. (2022). Remote vascular interventional surgery robotics: A literature review. Quant. Imaging Med. Surg..

[3] Matsui Y., Kamegawa T., Tomita K., Uka M., Umakoshi N., Kawabata T., Munetomo K., Iguchi T., Matsuno T., Hiraki T. (2024). Robotic systems in interventional oncology: A narrative review of the current status. Int. J. Clin. Oncol..

[4] Zhang L., Guo S., Yu H., Song Y., Tamiya T., Hirata H., Ishihara H. (2018). Design and performance evaluation of collision protection-based safety operation for a haptic robot-assisted catheter operating system. Biomed. Microdevices.

[5] Xia S., Zhu H., Liu X., Gong M., Huang X., Xu L., Zhang H., Guo J. (2019). Vessel segmentation of X-ray coronary angiographic image sequence. IEEE Trans. Biomed. Eng..

[6] Mehmood R., Iqbal N., Tahir A., Riaz M.M., Nawaz R. (2017). Real Time 3D Representation and Tracking of Guidewire for Image Guided Cardiovascular Interventions. GeNeDis 2016: Geriatrics.

[7] Sarmah M., Neelima A., Singh H.R. (2023). Survey of methods and principles in three-dimensional reconstruction from two-dimensional medical images. Vis. Comput. Ind. Biomed. Art.

[8] Jianu T., Huang B., Nguyen H., Bhattarai B., Do T., Tjiputra E., Tran Q., Berthet-Rayne P., Le N., Fichera S. Guide3D: A Bi-planar X-ray Dataset for Guidewire Segmentation and 3D Reconstruction. Proceedings of the Asian Conference on Computer Vision.

[9] Kim J., Nguyen P.B., Kang B., Choi E., Park J.-O., Kim C.-S. (2019). A novel tip-positioning control of a magnetically steerable guidewire in sharply curved blood vessel for percutaneous coronary intervention. Int. J. Control Autom. Syst..

[10] Ranftl R., Bochkovskiy A., Koltun V. Vision transformers for dense prediction. Proceedings of the IEEE/CVF International Conference on Computer Vision.

[11] Chen C.F.R., Fan Q., Panda R. Crossvit: Cross-attention multi-scale vision transformer for image classification. Proceedings of the IEEE/CVF International Conference on Computer Vision.

[12] Wang W.J., Tang B., Gu Z.H., Wamh S. (2025). Overview of Multi-View 3D Reconstruction Techniques in Deep Learning. Comput. Eng. Appl..

[13] Qi C.R., Su H., Mo K., Guibas L.J. Pointnet: Deep learning on point sets for 3d classification and segmentation. Proceedings of the IEEE Conference on Computer Vision and Pattern Recognition.

[14] Burgner J., Herrell S.D., Webster R.J. Toward fluoroscopic shape reconstruction for control of steerable medical devices. Proceedings of the Dynamic Systems and Control Conference.

[15] Hoffmann M., Brost A., Koch M., Bourier F., Maier A., Kurzidim K., Strobel N., Hornegger J. (2015). Electrophysiology catheter detection and reconstruction from two views in fluoroscopic images. IEEE Trans. Med. Imaging.

[16] Wagner M., Schafer S., Strother C., Mistretta C. (2016). 4D interventional device reconstruction from biplane fluoroscopy. Med. Phys..

[17] Breininger K., Hanika M., Weule M., Kowarschik M., Pfister M., Maier A. (2019). 3D-reconstruction of stiff wires from a single monoplane X-ray image. Bildverarbeitung für die Medizin 2019: Algorithmen–Systeme–Anwendungen. Proceedings des Workshops vom 17. bis 19. März 2019 in Lübeck.

[18] Jianu T., Huang B., Berthet-Rayne P., Fichera S., Nguyen A. (2023). 3D Guidewire Shape Reconstruction from Monoplane Fluoroscopic Images. Proceedings of the International Conference on Robot Intelligence Technology and Applications.

[19] Wang Y., Luo Y., Zu C., Zhan B., Jiao Z., Wu X., Zhou J., Shen D., Zhou L. (2024). 3D multi-modality Transformer-GAN for high-quality PET reconstruction. Med. Image Anal..

[20] Zhao L., Pang S., Chen Y., Zhu X., Jiang Z., Su Z., Lu H., Zhou Y., Feng Q. (2023). SpineRegNet: Spine Registration Network for volumetric MR and CT image by the joint estimation of an affine-elastic deformation field. Med. Image Anal..

[21] Yin G., Li P., Wei Z., Liu G., Yang Z., Zhao L. (2020). Magnetic dipole localization and magnetic moment estimation method based on normalized source strength. J. Magn. Magn. Mater..

[22] Paquet L., Solignac A., Koon K.T.V., Ohta M., Tsuruoka N., Haga Y., Fermon C., Pannetier-Lecoeur M., Ducharne B. (2025). Magnetic tracking for catheterization procedure, using giant-magnetoresistance and space-varying magnetic field free point. Sens. Actuators A Phys..

[23] Zhao Y., Shi L., Wei W., Xiao N. Intraoperative 3D shape estimation of magnetic soft guidewire. Proceedings of the 2025 IEEE International Conference on Robotics and Automation (ICRA).

[24] Liu Y., Liu L., Guo Y., Lew M.S. (2018). Learning visual and textual representations for multimodal matching and classification. Pattern Recognit..

[25] Bhat S.F., Alhashim I., Wonka P. Adabins: Depth estimation using adaptive bins. Proceedings of the IEEE/CVF Conference on Computer Vision and Pattern Recognition.

[26] Ramamonjisoa M., Lepetit V. Sharpnet: Fast and accurate recovery of occluding contours in monocular depth estimation. Proceedings of the IEEE/CVF International Conference on Computer Vision Workshops.

[27] Ranftl R., Lasinger K., Hafner D., Schindler K., Koltun V. (2020). Towards robust monocular depth estimation: Mixing datasets for zero-shot cross-dataset transfer. IEEE Trans. Pattern Anal. Mach. Intell..

[28] Bhat S.F., Birkl R., Wofk D., Wonka P., Müller M. (2023). Zoedepth: Zero-shot transfer by combining relative and metric depth. arXiv.

